# Philadelphia Hospital, Blockley

**Published:** 1845-01

**Authors:** 


PHILADELPHIA HOSPITAL, (BLOCKLEY.)

We understand the number of students in attendance on the Clinical Lectures, the present season, is 290: which is probably the largest class ever attached to any similar institution in this country. From the great number of patients who attend to be prescribed for at the University and Jefferson College, and the interest manifested by students in these *additional clinics*, it has been apprehended by some who are but partially informed on the subject, that the Hospital clinics would be superseded by those of the Colleges. This, we are convinced, was never designed by any one, and we are happy to be able to refer to the fact stated above, which conclusively shows that such is not the effect. We believe it is the wish of all who are engaged in teaching at the Colleges to encourage students to avail themselves, as far as practicable, of *all* the means afforded for clinical instruction in this city, and we are glad to perceive that, very generally, these advantages, great and numerous as they are, are appreciated.

---

PHILADELPHIA MEDICAL SOCIETY.

